# Effects of a Randomised Controlled School-Based Health Promotion Intervention on Obesity Related Behavioural Outcomes of Children with Migration Background

**DOI:** 10.1007/s10903-016-0460-9

**Published:** 2016-07-07

**Authors:** Susanne Kobel, Christine Lämmle, Olivia Wartha, Dorothea Kesztyüs, Tamara Wirt, Jürgen M. Steinacker

**Affiliations:** Division of Sports and Rehabilitation, Department of Internal Medicine II, Ulm University Medical Centre, Frauensteige 6, Haus 58/33, 89075 Ulm, Germany

**Keywords:** Child, Obesity, prevention and control, Diet, Sedentary lifestyle

## Abstract

Children with migration background (MB) appear to be at higher risk of developing obesity, therefore, prevention is necessary to avoid possible health inequalities. This study investigated a 1-year intervention with focus on increasing physical activity (PA) and fruit and vegetable intake (FVI), decreasing screen media use (SMU) and soft drink consumption (SDC) in children with MB. 525 children (7.1 ± 0.7 years) with MB who participated in the cluster-randomised study were assessed at baseline and after 1 year. Daily SMU, PA behaviours, SDC and FVI were assessed using a parental questionnaire. After one year, significant effects were found in the intervention group for FVI (*p* ≤ 0.035). Partially strong tendencies but no significant differences were found for PA and SDC. Although the effects are small, the intervention seems to reach children with MB. An intervention lasting longer than one year might result in more changes.

## Background

Obesity in combination with physical inactivity is one of the leading public health challenges in recent years [1]. In developed countries, the prevalence of childhood overweight and obesity has been increasing dramatically during the last decades [2]. Although current evidence suggests obesity rates are plateauing in a few countries [3], it has been shown that once children are overweight it is most difficult to treat or even reverse [4]. It long has been recognised that numerous health problems in childhood and later life are associated with obesity [5] and besides genetic predispositions the main reasons of this worldwide increase are behavioural and cultural factors [6, 7].

Although, the World Health Organisation (WHO) recommends an engagement of at least 60 min daily in moderate to vigorous physical activity (MVPA) for children [8], decreased physical activity levels from early childhood into adolescence and adulthood are well documented [9–11]. On the other hand, prolonged periods of sitting and a sedentary lifestyle rise continuously [9, 12]. Even at an early age, extensive use of screen media as one aspect of sedentary behaviour dominates many children’s day to day routines [13]. Paired with poor dietary behaviours, often consisting of a regular consumption of sugar-sweetened drinks as well as low fruit and vegetable intake, these are the most consistent factors associated with childhood overweight and obesity [14].

Moreover, children with migration background appear to be at higher risk of developing overweight and obesity [15, 16], have a higher sugar intake—assumingly due to a higher consumption of sweets—as well as a higher risk of low physical activity [17]. It has been suggested that this increased risk of overweight and obesity in children with migration background is a consequence of acculturation and lifestyle changes, such as giving up traditional dietary patterns and adopting new food habits as well as a high media exposure [18].

Children’s dietary and physical activity behaviours are mostly influenced by their homes [19]. In order to reach parents and promote a healthy lifestyle, education settings are promising for interventions as most children can be reached and already existing infrastructure can be used to support the implementation of interventions. Research shows that successful interventions should be integrated in the school curriculum and include aspects such as healthy eating and physical activity [20]. However, health promotion solely for children with migration background in a school-based setting is hardly possible and—especially since a holistic approach on health is being assumed—undesirable.

In 2009, the health promotion programme “Join the Healthy Boat” was implemented in primary schools in the south-west of Germany (federal state Baden-Württemberg). The materials and contents of the programme are designed for all primary school children and are fully incorporated in the curriculum whereby the focus lies on behaviour change to be more physically active, have a healthier diet and spend less time with screen media; all delivered through the classroom teacher but with substantial parental involvement, including parents’ nights, regular written information and so-called family homework (for more detailed information see [21]). In order to also reach parents with migration background, parent information was also delivered in Turkish and Russian, which are the two largest groups of migrants in Germany (28 and 30 %, respectively) [22].

Given that intervention effects can vary between populations with different ethnic backgrounds [23, 24] and therefore, the success of a health promotion programme could be influenced by ethnic or cultural factors, it is essential to investigate whether the implementation and intended outcomes of this intervention were achieved even in children with migration background. Hence, a large-scale evaluation study was carried out to examine children’s health behaviours after a one year intervention in respect of the programme’s main aspects: increased physical activity, decreased screen media use as well as a healthy diet including sufficient fruit and vegetable intake and a reduced consumption of sugar-sweetened drinks. This work reports intervention results of children with migration background only, intervention results of the general population are published elsewhere [25].

## Methods

### Intervention and Evaluation Design

“Join the Healthy Boat” is a school-based intervention to promote a healthy lifestyle in children. Key aspects of the intervention are more physical activity, less time with screen media and a more healthy diet, especially targeting a reduction of soft drink consumption and an increase of fruit and vegetable intake. The ready to use materials the teachers are given include one lesson per week (on physical activity, diet or screen media use) and daily exercise breaks of 10–15 min. The main focus lies on the promotion of healthy and active alternatives, which children are offered to choose in order to lead a healthier lifestyle. In order to enable children to carry home the learnt information, parents’ nights, regular parents’ letters and so-called family homework are provided; the latter require joint efforts of parents and child to solve the given exercises. Further information on development, materials, implementation and recruitment of teachers and pupils can be found elsewhere [21].

The evaluation of this health promotion programme was designed as a prospective, stratified, cluster-randomised, and longitudinal study with control and intervention group. Baseline measurements were taken prior to the programme’s start at the beginning of the academic year within 6 weeks in autumn, after that the intervention group integrated the programme into the curriculum whereas the control group stuck to the regular school curriculum. In autumn the following year, follow-up measurements were taken. Approval for the study was obtained from the University’s Ethics Committee, the Ministry of Culture and Education and was provided in accordance with the declaration of Helsinki.

### Participants

1943 first and second grade schoolchildren in 154 classes (80 classes in the intervention group; 74 classes in the control group) were assessed at baseline and 1736 of them at follow-up. A sub-sample of 525 (27 %) children (7.1 ± 0.7 years; 51.4 % female; 318 in the intervention group, 207 in the control group) was classified as having a migration background. Children were included in the sub-sample if at least one parent was born abroad or the child was spoken to in another language than German in the first 3 years of life. Prior to data collection, parents provided written informed consent to taking part in the study. Children gave their assent.

### Measures

Children’s height (cm) and body weight (kg) were assessed by trained staff to ISAK-standards [26] using a stadiometer and calibrated electronic scales (Seca 213 and Seca 826, respectively, Seca Weighing and Measuring Systems, Hamburg, Germany). Children’s body mass index (BMI) was calculated (kg/m^2^) and converted to BMI percentiles (BMIPCT) based on German reference data [27] in order to classify children into overweight (above the 90th percentile) and obese children (above the 97th percentile).

Daily screen media use, physical activity behaviours, soft drink consumption, daily fruit and vegetable intake at school as well as parental education levels (determined by mother’s and father’s highest education) and weight status were assessed using a parental questionnaire. The included questions are validated questions from the German Health Interview and Examination Survey for Children and Adolescents (KiGGS), which recently assessed health behaviour in 18,000 German children and adolescents [28]. Since parents confirmed on the consent form that they understand German as a language, no translations were used.

### Data Analysis

Statistics were performed using SPSS Statistics 21 (SPSS Inc., Chicago, IL, US) with a significance level set to α < 0.05. Descriptive statistics were calculated (mean values and standard deviations for continuous variables; frequencies and percentages for categorical variables). For the detection of group differences at baseline for categorical data, Fisher’s exact test was used. For group differences at baseline and follow-up, Chi-Square test was used. For inference statistical analysis, physical activity was dichotomised by reaching the physical activity guideline [8] or not, i.e. engagement on most days per week (4 days or more) of at least 60 min of MVPA. Based on recommendations of the American Academy of Paediatrics [29], screen media use (TV, PC and game consoles) was dichotomised using a cut-off point of 1 h per day. Soft drink consumption was dichotomised by consuming soft drinks more than once versus less than once per week (median split). The frequency of fruit and vegetable intake at school was assessed as “always”, “often”, “rarely” and “never” and dichotomised in always/often versus rarely/never. Subsequently, logistic regression was used to determine odds ratios (OR) for all health outcomes, controlling for baseline, age, weight status and parental education level. Fruit and vegetable intake at school was additionally analysed using a related-samples marginal homogeneity test, for control and intervention group, respectively.

## Results

There were no significant gender differences or significant differences between the participants in the control group compared to the intervention group with regards to gender, age, height, body weight, BMIPCT, weight status as well as parental education level and weight status. Participants’ characteristics at baseline and follow-up are shown in Tables 1 and 2. The prevalence of overweight children with migration background (including obesity) is 11.8 % and of obese children with migration background alone 5.7 %.

**Table 1:** Baseline characteristics of participants with migration background

	Missing values	Intervention	Control	Total
		(n = 318)	(n = 207)	(n = 525)
Age, years [m (SD)]		7.15 (0.66)	7.08 (0.66)	7.12 (0.66)
Boys, n (%)		149 (46.9)	106 (51.2)	255 (48.6)
Anthropometry				
BMI, kg/m^2^ [m (SD)]	18	16.50 (2.53)	16.11 (2.13)	16.34 (2.39)
BMIPCT [m (SD)]	18	54.70 (29.07)	50.94 (27.59)	53.20 (28.53)
Overweight and obesity, n (%)	18	42 (13.8)	18 (8.9)	60 (11.8)
Parental characteristics				
Tertiary family educational level, n (%)	38	71 (23.9)	46 (24.2)	117 (24.0)
Overweight (mother), n (%)	41	103 (35.0)	66 (34.7)	169 (34.9)
Overweight (father), n (%)	67	181 (64.9)	109 (60.9)	290 (63.3)
Health and lifestyle characteristics				
MVPA on ≥4 days/week ≥60 min/day, n (%)	45	69 (23.6)	54 (28.7)	123 (25.6)
Screen media ≥1 h/day, n (%)*	5	83 (26.3)	43 (21.1)	126 (24.2)
Soft drinks ≥1 time/week, n (%)	6	100 (31.6)	69 (34.0)	169 (32.6)
Fruit and vegetable intake at school often/always, n (%)	25	229 (75.3)	156 (79.6)	385 (77.0)

*m (SD)* mean (standard deviation), *BMI* body mass index, *BMIPCT* BMI percentiles, *MVPA* moderate to vigorous physical activity

* Significant difference between control and intervention group

**Table 2:** Follow-up characteristics of participants with migration background

	Missing values	Intervention	Control	Total
		(n = 318)	(n = 207)	(n = 525)
Anthropometry				
BMI, kg/m^2^ [m (SD)]	46	16.99 (2.84)	16.53 (2.41)	16.81 (2.68)
BMIPCT [m (SD)]	46	55.05 (30.09)	50.27 (28.32)	53.18 (29.47)
Overweight and obesity, n (%)	46	44 (15.1)	19 (10.2)	63 (13.2)
Health and lifestyle characteristics				
MVPA on ≥4 days/week ≥60 min/day, n (%)	126	64 (26.2)	42 (27.1)	106 (26.6)
Screen media ≥1 h/day, n (%)	108	49 (19.4)	30 (18.2)	79 (18.9)
Soft drinks ≥1 time/week, n (%)	109	67 (26.6)	44 (26.8)	111 (26.7)
Fruit and vegetable intake at school often/always, n (%)*	130	190 (80.2)	125 (79.1)	315 (79.7)

*m (SD)* mean (standard deviation), *BMI* body mass index, *BMIPCT* BMI percentiles, *MVPA* moderate to vigorous physical activity

* Significant difference between control and intervention group

### Physical Activity

When first assessed, children with migration background engaged in 60 min of MVPA on 2.7 (±1.8) days per week. Moreover, 28.4 and 23.0 % of boys and girls, respectively, spent 4 days or more per week in MVPA for at least 60 min. A total of 6.0 % of children with migration background spent a minimum of 60 min being moderately to vigorously physically active on 7 days per week, as recommended by the WHO [8]. At baseline, no differences between control and intervention group were found. However, boys displayed significantly higher activity levels than girls [60 min on 2.9 (±1.9) vs. 2.5 (±1.7) days, respectively; *p* = 0.004].

At follow-up, the number of days children with migration background engaged in 60 min of MVPA remained the same (2.7 ± 1.7 days per week), yet one third of boys and 20.1 % of girls, engaged at least 4 days per week in MVPA for at least 60 min. Further, 5.0 % of children with migration background reached the recommended 60 min of MVPA on 7 days per week. Also, after one year, no significant intervention effects could be shown regarding the amount of physical activity between control and intervention group (OR 1.085 [0.622; 1.892], *p* = 0.775).

However, there is a tendency towards a decrease in physical activity in the intervention group. Although a far greater reduction of physical activity in the control group could be observed (intervention group: children with migration background achieving recommended 60 min of MVPA daily: 5.5 % at baseline, 5.3 % at follow-up; control group: 6.9 % at baseline, 4.5 % at follow-up), statistical significance however, was not reached.

### Screen Media Consumption

At baseline, 25.8 and 22.8 % of boys and girls, respectively, spent a minimum of 1 h per day using screen media, including television, computer/laptop and video games. No gender differences could be observed, yet, at baseline, children with migration background in the intervention group showed significantly higher screen media use than those in the control group (*p* = 0.006).

After the intervention, the proportion of children with migration background using screen media for at least 1 h daily reduced to 20.8 % of boys and 17.2 % of girls, with no significant gender differences but also no significant group differences between intervention and control anymore (OR 0.932 [0.497; 1.747], *p* = 0.826). Nevertheless, screen media use decreased in both groups with a tendency towards a greater reduction of screen media use in the intervention group. Even though at baseline, children with migration background in the intervention group used significantly more screen media than those in the control group (intervention group: 26.3 % at baseline; 19.4 % at follow-up, control group: 21.1 % at baseline; 18.2 % at follow-up), statistical significance however, was not reached either.

### Soft Drink Consumption and Fruit and Vegetable Intake

Looking at children’s soft drink consumption, at baseline, 32.6 % of children with migration background consumed sugar-sweetened beverages at least once per week. There was no significant difference between control and intervention group, a significant gender difference however, with girls drinking less sugar-sweetened beverages could be observed at baseline (boys: 35.4 %, girls: 29.8 %; *p* = 0.007).

Similarly, even though, a reduction of soft drink consumption could be seen in both groups, at follow-up, there was no significant gender difference nor was there a difference between control and intervention group (OR 1.010 [0.587; 1.738], *p* = 0.973).

Data on children’s fruit and vegetable intake at school revealed that at baseline 2.9 and 20.6 % of children with migration background never or rarely eat fruit and vegetables during their break and lunch times, respectively. There was a significant gender difference with 19.2 % of girls and 27.1 % of boys never or rarely eating fruit and vegetables at school (*p* = 0.043).

**Fig. 1:**
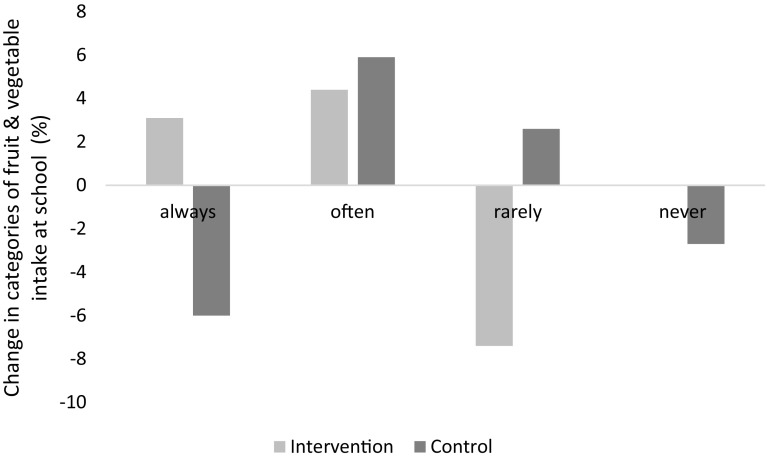
Changes (in percentage) in categories of fruit and vegetable intake at school between baseline and follow-up for intervention and control group

At baseline, no difference between control and intervention group was found; after 1 year of intervention, logistic regression analyses revealed no difference between the two groups (OR 1.663 [0.895; 3.090]; *p* = 0.108). Significant positive effects however could be observed when analysing data using a related-samples marginal homogeneity test with children with migration background in the intervention group eating fruit and vegetables significantly more often than children with migration background in the control group (children who never or rarely eat fruit and vegetables at school in the intervention group: 25.4 % at baseline, 18.0 % at follow-up; control group: 20.5 % at baseline, 20.5 % at follow-up; *p* = 0.035; Fig. 1).

## Discussion

The purpose of this study was to evaluate the effectiveness of a low-threshold, teacher-centred health promotion programme over one year on primary school children with migration background. Key focus areas of this intervention are an increase in daily physical activity, a reduction of screen media use and a more healthy diet, especially a reduction of sugar-sweetened beverages and an increased fruit and vegetable intake.

In this study, there was no significant intervention effect on children’s daily MVPA, which is consistent with other research [30]. Within one year, children’s activity levels decreased in both groups. An age induced decline of physical activity levels has been reported previously [31, 32], which is especially pronounced at school entry [33]. However, after one year, a trend towards a greater decrease of daily MVPA in the control group could be observed. Nevertheless, this intervention aimed at children changing their activity behaviour during their day to day lives without additional PE lessons, which may not necessarily result in a change of MVPA levels. The main focus of this programme lies on delivering alternatives to spend ones leisure time as well as daily routines more actively. Nevertheless, very similar results could be observed in the overall “Healthy Boat” group (children with and without migration background) [25], suggesting that the physical activity aspect of this intervention might be too low-threshold. However, as suggested by Vander Ploeg and colleagues [34] more positive effects might have been achieved with a longer lasting and more intense intervention.

Further, the notably low levels of physical activity in this study are reflected in previous research [35, 36] showing that especially Turkish children engage in very little physical activity [37].

But not only low physical activity levels are higher in children with migration background, other risk factors for overweight, especially television watching has been shown to have a particularly high influence [16, 38]. In this study, no significant intervention effects were seen with regards to screen media use. After one year, television viewing and computer use decreased slightly in both groups, with a tendency of a greater reduction in the intervention group. Similar effects have been reported previously [39, 40] whereas parental involvement was identified as one of the most important aspects for a successful intervention [41]. The present programme targeted screen media use solely by offering children active alternatives for sedentary behaviour. Parents were informed about these alternatives as well as about recommendations and age-appropriate time limits for screen media use by letters and family homework, which includes a “screen-free weekend” with the whole family, offering action alternatives for an active weekend. Although all letters to parents were also provided in Turkish and Russian, parents from other countries may have not understood the given information. This would be further supported by the fact that children without migration background of the whole cohort showed a significant decrease in screen media use after one year [25]. Therefore, this aspect clearly failed to reach children with migration background (and their families). However, it has been suggested that a change in children’s behavioural capability precedes an actual behaviour shift [42], which might imply that one year for a very low-dose intervention as this one is not sufficient to achieve effects on children’s screen media use.

The diet component of this intervention focussed on a reduction of sugar-sweetened beverages and a regular intake of fruit and vegetables. It has been shown that soft drink and juice consumption differs in children with migration background compared to German children, with children with migration background drinking more sugar-sweetened beverages [43] and that these differences may modify intervention effects of health promotion programmes [44]. In accordance with recent research [25, 45], no intervention effect was observed with regards to the amount of sugar-sweetened drinks children consumed per week. This may be due to the already low levels of soft drink consumption (one drink per week) or the intervention method since soft drink consumption was addressed to parents using letters only.

Regular fruit and vegetable consumption however, was communicated using family homework and different hands-on exercises, such as healthy snacks at school, joint healthy breakfast at school and at home, which also required parental involvement. The important role parents play in the development of healthy eating behaviours is indisputable [46] and nearly a quarter of children in this study never or rarely ate fruit and vegetables at school. But on the other hand, nearly 40 % of boys and girls took some piece of fresh fruit or vegetable to school every day (data not shown), which is comparable with other German data of 15,865 children (3–17 years) on fruit and vegetable intake, showing that 47 % or boys and 55 % of girls eat fruit at least once per day [43].

In the present study, significant positive intervention effects could be observed for fruit and vegetable intake at school, even though at baseline, children with migration background in the control group ate fruit and vegetables more regularly than their counterparts in the intervention group. This could possibly be due the fact that one focus of the programme lies on fresh fruit and vegetables as part of a healthy breakfast and snack at school. Parents were informed about the importance of daily fruit and vegetable intake for their children’s health as well as being given ideas and little recipes for healthy snacks. At school, healthy breakfasts and snacks have been discussed and prepared together. Further, a recent study in the US showed that getting children to try different kinds of fruits and vegetables can increase their liking and therefore their intake of those [47]. This might suggest that the action alternatives this intervention offers may well lead to children getting to know and even like certain foods they have not tried before. Especially children with migration background could benefit from these methods since they might get to know foods which are unusual in their families and culture. Moreover, recipes and pictures of fruit and vegetables are easily understood even without fluency. This would also be supported by the fact that when considering children without migration background of the same intervention no effects with regards to fruit and vegetable consumption could be reported (data not shown).

However, there are some limitations to be considered when interpreting these results. The subjective assessment methods of all three variables (physical activity, screen media use, diet) and the potential recall bias and social desirability are a limitation of this study. Furthermore, language might for parents with migration background have been a barrier when answering questions about their children and themselves. This might have led to a misinterpretation of some questions. Additionally, changes in health behaviours from interventions targeting children usually occur in the long term [48], which would mean one year of intervention—especially as very low-threshold one like this one—may not have been long enough to detect behaviour changes. Nevertheless, the large sample size and the randomised controlled design with a control group are major strengths of this study. Still, generalisation of these results is not possible and further detailed research in this area is necessary—especially in order to understand mode of action and barriers for the here targeted health behaviours.

## Conclusions

The evaluation of a very low-threshold teacher-centred health promotion programme has shown the ability to significantly increase fruit and vegetable intake in children with migration background as well as tendencies in other targeted areas, namely more physical activity and reduced screen media use. Although some effects are small, the intervention—especially its action alternatives and parental involvement—seems to reach children with migration background and their parents. It is especially important to look at this group since it has been shown that children with migration background differ from those without migration background in a number of health-related behaviours such as physical activity, diet and drinking habits [16, 49] as well as being of higher risk of overweight [15, 50]. So far only few prevention or health promotion programmes exist for these populations which are generally less effective [51–53] and because most health related behaviours are hard to change within the space of one year, longer and more intense interventions are necessary. However, healthcare professionals should be aware of the low levels of physical activity and increased consumption of sugar-sweetened beverages among children with migration background. Further studies on why some aspects of this intervention only showed effects in children without migration background are required in order to modify those. Additionally, more research on the level of parental involvement and their barriers, particularly for parents from a migration background, would be of interest to be able to better tailor health promotion programmes for this group and to avoid a potential broadening of health inequalities.

## New Contribution to the Literature

Children with migration background appear to be at higher risk of developing overweight and obesity; therefore, early and effective prevention programmes are necessary in order to avoid a possible expansion of health inequalities.

This theory-based, teacher-centred intervention focussed on increased physical activity and fruit and vegetable intake as well as decreased screen media use and soft drink.

Findings show that only 5 % of children with migration background reached the recommended 60 min of moderate to vigorous physical activity on 7 days per week. After a 1-year intervention however, there is a tendency towards a (age-related) decrease in physical activity in the intervention group, whereas a far greater reduction of physical activity in the control group could be observed.

Also, after the intervention, screen media use decreased in both groups with a tendency towards a greater reduction of screen media use in the intervention group. Even though at baseline, children with migration background in the intervention group used significantly more screen media than those in the control group.

Significant positive effects however could be observed with children with migration background in the intervention group eating fruit and vegetables significantly more often than children with migration background in the control group.

These findings are unique in their nature since to date only limited and hardly comparable data are available on physical activity and dietary behaviours of children with migration background in Germany. Especially, the large sample size and the prospective, stratified, cluster-randomised and longitudinal study design with an intervention and a control group are strengths of this research.
